# Pleasant for some and unpleasant for others: a protocol analysis of the cognitive factors that influence affective responses to exercise

**DOI:** 10.1186/1479-5868-7-15

**Published:** 2010-02-07

**Authors:** Elaine A Rose, Gaynor Parfitt

**Affiliations:** 1 School of Physical Education, University of Otago, PO Box 56, Dunedin, 9054, New Zealand; 2 School of Sport and Health Sciences, St. Luke's Campus, University of Exeter, Heavitree Road Exeter, EX1 2LU, UK

## Abstract

**Background:**

At exercise intensities around ventilatory threshold (VT), the extent to which individuals experience pleasure or displeasure from the exercise varies between individuals. One source of this variability is proposed to be the cognitive appraisal that occurs during the exercise which influences the generation of the affective response. When individuals self-select their own intensity they choose to exercise around VT and experience more positive affective responses, again the explanation being that cognitive appraisal processes influence the choice of intensity and resulting affective response. However, the specific factors that comprise this appraisal process have not been thoroughly explored. In addition, it is not clear if activity status influences this appraisal and different cognitive factors play a role in the generation of affective responses. Therefore, the purpose of this study was to explore the cognitive factors that influence the affective response experienced during prescribed and self-selected intensity exercise in low-active and high-active women.

**Methods:**

Seventeen low-active and 15 high-active women (*M *age = 45 years, *SD *= 10) completed a graded exercise test and two 30 min bouts of treadmill exercise, one at a self-selected intensity and one prescribed at an intensity around VT. Using 'think aloud' procedures, every five min, the women were asked to provide an affective response and explain the thought processes that caused them to report that affective response. Using inductive content analysis, the verbal reports provided by the women were analysed for key themes and categories that emerged as explaining the factors that underpinned the generation of the affective response. Data from the low-active and high-active women were analysed separately.

**Results:**

Concepts relating to pre-exercise affective state, perceptions of ability, immediate and anticipated outcomes, attentional focus and perceptions of control emerged. The physiological demands of the exercise stimulus influenced affective responses through themes related to the interpretation of physiological symptoms and the physiological state of the body. No thematic differences emerged between high-active and low-active women.

**Conclusion:**

Results highlight the complex interaction of psychological and physiological influences in producing an affective response to exercise and provide insight into how exercise can be structured to elicit positive affective responses.

## Background

The Hedonic principle suggests that individuals choose to participate in behaviours which are pleasant (lead to positive affective responses) and avoid those that are unpleasant [1]. The role of affective responses in influencing future behavioural decisions extends to exercise. Williams et al. [2] have shown that affective responses to a moderate intensity exercise stimulus predicted exercise behaviour 6 and 12 months later. One of the key determinants of the affective response to exercise is the intensity at which it is performed. To understand the relationship between exercise intensity and affective responses research has focused on quantifying pre- to during and pre- to post-exercise changes in affective responses at different exercise intensities [3-14]. One of the key findings of this research is that there are great inter-individual differences in the affective response to exercise, particularly at intensities classified as 'moderate' and also those classified as lying around the ventilatory threshold (VT). However, except for research investigating the role of self-efficacy and attentional focus, little attention has been focused on understanding the cause of the affective changes during exercise or the cause of the individual differences in affective response. Rose & Parfitt [12] suggested that "knowing 'why' someone feels the way he or she does during exercise...could be just as important as knowing 'how' he or she feels" (p. 306) and could provide significant theoretical and practical advances [3].

A growing body of literature grounded in the proposals of the dual-mode model (DMM) [15,16] has demonstrated consistent results when investigating affective responses during and following exercise of different intensities at a group level. An underlying premise of the DMM is that exercise intensity should be defined according to a fixed metabolic profile (such as ventilatory threshold or lactate threshold) rather than according to a percentage of maximal capacity. This ensures that individuals are in the same metabolic state when responding affectively to the exercise, a situation that cannot be guaranteed when using %HR_max _or %VO_2max _[17]. Using a fixed metabolic marker removes a potential confound when comparing affective responses between individuals and between intensities. Using VT to define exercise intensity, research has shown that during intensities prescribed below and around VT affective responses have a mainly positive valence [4,5,9,11,12,14]. In comparison, during exercise prescribed at an intensity above VT, affective valence is less positive and in some cases negative, especially when exercise continues until volitional exhaustion [4-6,8,11,12,14]. What is more interesting however, is that for intensities below VT and especially around VT, great variability exists between individuals in terms of whether exercise will induce an increasing, decreasing or stable sense of pleasure [e.g., [12]]. Whereas, above VT there is less variation between individuals and generally everyone experiences a decline in pleasure. The DMM proposes that the interplay between two factors explains these affective responses to exercise; a cognitive appraisal process and interoceptive cues. At intensities below VT, the affective response to exercise is generated mainly through a cognitive appraisal process (hence the variability in responses that occur). As intensity increases to sit around VT, the cognitive appraisal process remains dominant but there is also some influence from the interoceptive cues generated from the symptoms of the exercise. As intensity continues to increase beyond VT, the interoceptive cues become the sole influence and override the influence of cognitive processes (producing the homogenous negative affective response).

As a means of reducing these individual differences, researchers adopted self-selected intensity protocols [7,10-12,18]. Rose and Parfitt [12] proposed that allowing an individual to self-select their own exercise intensity should result in more positive and less variable affective responses because the individual's cognitive appraisal and resultant affective response will influence their intensity selection to maintain or enhance the affective response. Interestingly, on average, individuals choose to exercise at intensities around VT [10-12,19] but individual differences in the chosen intensity are also demonstrated. Although affective responses during self-selected paradigms are in the positive range, individual variability, although reduced, is not eliminated [e.g., [12]].

Ekkekakis [15] speculated that self-efficacy, prior exercise history, goal achievement, personality characteristics and the social context would be involved in the cognitive appraisal process. Research has since supported some of these cognitions as important to the affective response. Ekkekakis et al. [5] have shown that the more predisposed an individual is to tolerate the symptoms of the exercise and continue to exercise, and the extent to which individuals have a preference for the imposed intensity, predicted 16-18% and 16-21% of the variance in affective responses towards the end of exercise at intensities around VT. Furthermore, quantitative research has consistently shown that there is a relationship between self-efficacy and the affective response to exercise [e.g., [20,21]]. Associative and dissociative thought patterns during exercise have also shown a relationship with affective responses [e.g., [14,22,23]]. Blanchard et al. [22] found that having dissociative-external (non-exercise related stimuli not related to one's self) thoughts while running resulted in improved affect pre- to post-exercise. Welch et al. [14] found that at the beginning of a graded exercise test their female participants had a largely dissociative attentional focus and as the exercise intensity increased their attentional focus became progressively more associative and was accompanied by a progressive decline in affective responses. Interestingly, they reported that at VT, attentional focus was halfway between fully associative and fully dissociative suggesting that participants were thinking about the physical sensations of the exercise as much as they were able to focus on non-exercise related stimuli.

More recently, using a qualitative methodology, Rose and Parfitt [12] investigated the factors that influenced the generation of affective responses during exercise below, at and above VT and to a self-selected intensity. They concluded that to achieve a positive affective response individuals need to experience a combination of the following factors: to perceive that they have the ability to cope with the intensity and complete the exercise session, to feel they are being comfortably challenged and that the exercise is not out of their control, to perceive they are gaining benefit from the exercise and to be able to dissociate from the symptoms of the exercise. The factors appeared to interact to influence the final affective response and the importance of each factor differed between exercise intensities and between participants as a result of their unique cognitive appraisal. Results of this study supported the speculations of Ekkekakis [14] and also showed the significance of perceptions of control and different thought patterns on affective responses. Although this study was able to identify the cognitions that influenced an individual's affective response, it was limited in the fact that the themes were generated from an interview that asked individuals to recall how they had felt and what had caused them to feel that way after the exercise session had been completed. By employing this methodology, individuals may not have provided an accurate or full account of all the underlying processes that influenced their affective response at that time, or they may have drawn upon prior assumptions or theories about what they expect should have caused their response [24].

The use of protocol analysis [25] to elicit verbal reports of what participants think about while they are exercising will overcome the limitations of the retrospective recall approach. This process, where participants 'think aloud' while completing a task, has been shown to elicit an accurate verbal report of the cognitive processes going on at the time and avoids the need for any additional cognitive processing to generate explanations [25]. Importantly, Ericsson and Simon [26] state that if the information contained within the working memory includes factors relating to focus of attention, self commands, current sensations or thoughts or previous activities then participants should be able to think aloud concurrent with performing a task. Protocol analysis has been used extensively in studies where participants are asked to undertake a problem solving exercise (e.g., a mathematical problem) and verbalise their attempts to solve the problem [e.g., [27,28]]. More applicably, it has also been used to examine dissociative/associative thought processes that runners have during exercise [22,29]. For example, Blanchard et al. [22] asked individuals to "talk about anything on their minds" (p.124) in the last 5 minutes of a 20 minute run. Thus, protocol analysis procedures have been used successfully to elicit the cognitive processes underlying the pattern of associative and dissociative thoughts during running and would seem to be an appropriate procedure to investigate the cognitive processes underlying the generation of an affective response.

The extent to which the cognitive appraisal processes differ according to activity or fitness status has not been examined. Quantitative research has shown that activity or fitness status influences the affective responses generated by bouts of exercise at different intensities [e.g., [30-33]]. Since activity status seems to impact affective responses at different intensities, the cognitive appraisal process that helps create the affective response may also be different between high-active and low-active participants. For example, it would be expected that high-active participants would have higher perceptions of ability that may lead them to experience more positive affect, particularly at higher intensities. The knowledge of how high-active individuals experience pleasure from exercise may provide insight into how exercise can be structured to elicit a more positive affective response for low-active participants.

In light of previous research, the purpose of this study was to investigate the cognitive factors that influence the generation of an affective response to exercise during exercise conducted at VT and at a self-selected intensity in both low-active and high-active women using protocol analysis.

## Methods

### Participants

Nineteen low-active and 17 high-active women volunteered to participate by responding to adverts placed in a local newspaper, the University newsletter, local gyms and on hospital noticeboards. Two low-active and two high-active women dropped out of the study due to injury sustained while not involved in the study. Therefore, 17 low-active (*M *age = 43.9 years, *SD *= 9.7) and 15 high-active women (*M *age = 46.4 years, *SD *= 10.6) completed the requirements of the study.

Women who were recruited as low-active were required to have not exercised more than once a week for the previous six months. Those who were recruited as high-active were required to have participated in at least three exercise sessions per week for 20 min or more at a moderate to vigorous intensity for the previous six months. Leisure Time Exercise Questionnaire [34], results showed the high-active women engaged in significantly greater amounts of physical activity per week than the low-active (*M *= 66.3 METS, *SD *= 23.19 compared to 17.6 METS, *SD *= 13.5). Furthermore, the high-active women had a significantly higher mean  (40.4 ml kg^-1 ^min^-1^, *SD *= 4.7) compared to the low-active (31.1 ml kg^-1 ^min^-1^, *SD *= 5.0). Based on the American College of Sports Medicine's [35] norms for VO_2max _for females aged 40-49, our high-active group lie between the 80-90^th ^percentile, while our low-active group lie between the 20-30^th ^percentile. Together, these results suggest that our classification of participants into high- and low-active is justified.

Before being accepted into the study, the women completed a medical history questionnaire which ensured that they did not have cardio-respiratory or muscular contraindications to participate in exercise and that they were not currently taking any medications for health problems. All participants gave written informed consent. Ethical approval was granted from the University Ethics Committee. This study is part of a larger project investigating affective responses to prescribed and self-selected exercise in low-active and high-active women. The quantitative data arising from the project has been submitted for publication.

### Measures

Concurrent verbal reports [25,26] were used to elicit the thoughts of the women during the exercise sessions. Using standardized prompts, this 'think aloud' method encouraged participants to report the thought processes that led them to verbalise a particular affective response immediately after that affective response had been provided. This method provides an indication of what the women were aware of during the exercise session and provides insight into the decision-making processes underlying the generation of their affective response [36]. Ericsson and Simon [25] stated, "verbal reports elicited with care and interpreted with full understanding of the circumstances under which they were obtained are a valuable and thoroughly reliable source of information about cognitive processes" (p. 247) particularly when the thoughts are verbalized immediately or very shortly after they are attended to. The procedure for the think aloud protocol is described below.

Affective responses were measured from the perspective of the circumplex model [37]. Affective valence (pleasure/displeasure) was measured using The Feeling Scale [38] where participants rate their current feelings on an eleven point bipolar scale ranging from +5 (very good) through 0 (neutral) to -5 (very bad). Perceived activation was measured using The Felt Arousal Scale of the Telic State Measure [39] where participants are asked to rate their levels of arousal on a six-point scale ranging from low arousal (1) to high arousal (6).

### Procedures

All 32 women completed two 30 min bouts of treadmill exercise on the Quinton Series 90 Q65 treadmill in a laboratory, during which they completed a concurrent verbal report of their thoughts during each session. One bout was performed at a prescribed intensity corresponding to the individual's ventilatory threshold (VT) and the other at a self-selected intensity (order counterbalanced). One exercise session was completed per week at the same time of the day and on the same day of the week. Prior to these exercise sessions, individuals participated in a maximal graded treadmill test to volitional exhaustion test protocol. This session served to establish their peak aerobic capacity () and VT from which the prescribed intensity was determined and the self-selected intensity could be calculated. The exercise test was based on the Balke-Ware treadmill graded exercise test protocol [35]. The women walked/jogged on a gradient of 0% for 3 min. The speed was then increased and every min thereafter, the gradient was increased by 1-2%, while maintaining a constant speed. The test continued until the participant reached the point of volitional exhaustion. Continuous breath analysis was performed using a Sensormedics 2900 Metabolic Cart (Sensormedics Corporation, Yorba Linda, Ca) metabolic analysis system and subsequently, the data was averaged over 20-s intervals and used in the determination of . The analyser was calibrated before each test. The test was classified as a maximal effort if two of the standard criteria for reaching maximal oxygen uptake were reached: 1) a peak or plateau in oxygen consumption (changes of less than 2 ml^-1 ^kg^-1^min with one incremental change in intensity), 2) a respiratory exchange ratio of ≥ 1.1 and 3) reaching or exceeding a final heart rate of age-related maximum (220 bpm - age).  was determined as the highest  value attained after reaching the standard criteria. The V-slope method was used to determine VT.  was plotted against  and from visual inspection of the graph, VT was determined to be the point at which the first disproportionate increase in  occurred [40]. Two investigators independently assessed the graphs and if there was disagreement between these two investigators, a third investigator analyzed the graphs and discussions between the three investigators were held until a consensus was reached on where VT lay. This visit also served as a familiarization session for the scales used to measure affective responses.

#### Experimental Exercise Sessions

After being fitted with a heart-rate monitor (Polar PE3000, Finland) a pre-exercise FS and FAS value was provided. The women then stood on the treadmill and were given the following standardized details on the purpose of the session and for the think aloud procedure,

The purpose of the session is to find out how you feel while you are exercising and what factors influence the way you are feeling while you are exercising. Every 5 min you will be asked to provide a measure of the Feeling Scale and the Felt Arousal Scale and I will ask you to provide an account of the thoughts that influenced your responses to those scales. I am interested in anything and everything that runs through your head in terms of how you are feeling and what is influencing the way you are feeling. If at any point through the exercise you feel a certain way or think 'that's why I'm feeling this way' then please verbalise those thoughts, you don't have to wait until the 5 min period is up.

A microphone was then fitted to the collar of the participant's t-shirt. The microphone was attached to a digital voice recorder (Olympus WS-100). The recorder was kept on 'pause' during the exercise session and at the end of each 5 min exercising period the recorder was turned to 'record' by the researcher. Additionally, if the participant started to speak and verbalise their thoughts at other points during the exercise session the recorder was again switched to record by the researcher.

In the prescribed intensity condition, the women were then told that they would be exercising for 30 minutes and that the intensity of the session would be set for them. They were given a 2 min warm-up period and then the intensity was set to the required intensity for a further 30 min. In the self-selected intensity condition, the women were told,

You will be exercising on the treadmill for 30 min, I would like you to select an intensity that you prefer and that you would consider doing regularly. You will have the opportunity to change the speed and the gradient every 5 min if you wish.

They were then given instructions on how to manipulate the speed and gradient of the treadmill and were told to play around with the intensity until they felt they had found the intensity that they preferred (typically around 1-2 min later) and then the 30 min exercise period started.

At the end of each 5 min period, the women were asked to provide a reading from the FAS and then FS scales that were pinned to the wall next to the treadmill. They were then encouraged to begin the think aloud procedure by the prompts, "What ran through your mind to help you decide that you felt that way?" Further probing questions were used to prompt the participant to elaborate on what they meant, to gain clarity in meaning and to ensure that all explanations for affective state were elicited. Examples of these probes include, "Can you tell me what you mean by good/fine/that?", "What information do you use to decide that you feel comfortable?", "How do you know the intensity is easy". Although this practice deviates from the true protocol analysis procedures and asks individuals for more detail than is contained in their recalled thoughts, this procedure was necessary to gain a complete understanding of the cognitive factors underpinning the affective response. Ericsson [41] reports that instructions to generate self-explanations for the content of the thoughts have been found to improve the participant's comprehension, memory and learning compared to merely thinking aloud. The rationale for not using such prompts is that some participants may have to go beyond any retrievable memory to provide an answer and will have engaged in additional cognitive processing to generate the thoughts which provide the required explanation. Ericsson [27] states that this changes the sequencing of mediating thoughts occurring during the problem solving task. However, in this study, the sequence of the mediating thoughts is not important since we are not determining the solution of a problem solving task, we are merely trying to understand all of the thoughts that have influenced the affective response. Thus the validity of the protocol analysis was not compromised.

### Analysis

The verbal reports generated during the exercise sessions were transcribed verbatim. These transcripts provided the documents from which a thematic analysis was undertaken. The aim of thematic analysis is to identify themes within the data, in this case, themes relating to the cognitive factors which influenced the generation of an affective response. Unlike content analysis, thematic analysis is an inductive process because the categories into which the themes will be classified have not been predetermined prior to the analysis [42]. To become fully immersed in the data, the transcripts were read (and checked) while listening to the original digital recording. Following this process, raw data quotes capturing a distinct thought were identified and given a conceptual label. These thoughts were then analyzed for commonality of meaning and organized into themes. Common themes were then combined into core categories. The themes and categories were compared and contrasted until no new themes could be determined and the data could be accounted for by the core categories [43]. The interview transcripts of the high-active and low-active women were analysed separately, the high-active data was analysed first. To ensure trustworthiness of the analysis, two researchers independently identified the raw data quotes and emergent themes and following discussion established the core categories [43]. Direct quotes have been provided so that readers can experience for themselves the women's perspectives [44]. The time during the exercise session at which each quote was recorded, whether it belongs to an high-active (HA) or low-active (LA) woman and whether it was taken from the prescribed (P) or self-selected (SS) condition are shown in brackets.

## Results and Discussion

To provide context to the qualitative data, across the 30 min, statistical analysis using two factor repeated measures analysis of variance showed that on average, the individuals exercised at the same intensity in the at-VT and self-selected conditions (results of the statistical analysis are available on request to the first author). Importantly, on average, the high-active and low-active women chose to exercise at the same intensity relative to their VT and as expected, this meant that the high-active women exercised at a significantly higher relative intensity than the low-active women (high-active *M *= 81.6% HR_peak_, *SD *= 8.1; low-active *M *= 68.7% HR_peak_, *SD *= 8.2).

The core categories that emerged as underlying the generation of affective responses in the high-active and low-active groups were similar, although there were differences in the themes which comprised the core categories. All categories and themes are shown in Table 1. To avoid repetition, the results from the two groups were merged and any differences in emergent themes found between the high-active and low-active women were explicitly identified and discussed. Since the average exercise intensity in both conditions was the same, data was not specifically analysed to investigate differences in the prescribed and self-selected conditions, however, the raw quotes that comprised each theme were examined to ascertain whether or not the theme was unique to one exercise intensity condition. If it was clear that a theme was more prevalent to one of the conditions, this was discussed. Importantly, the categories are not presented in any order of importance because protocol analysis does not determine which thought processes had greater or lesser importance to the generation of affective responses. Similarly, we do not present information on how frequently a particular theme or category was discussed because our underlying philosophy is that all the information provided, however frequent, is important to build the overall picture of the cognitive influences on affective responses [43].

**Table 1:** Factors influencing the affective responses of low and high active women.

Low-Active Group		High-Active Group
State of mind	Pre-Exercise Affective State	
+ve pre-exercise affective state		
-ve pre-exercise affective state		-ve pre-exercise affective state

Doing exercise	Outcomes of Doing Exercise	Doing exercise
Consequences of exercise		Consequences of exercise
Enjoyment of exercise		Enjoyment of exercise
Benefits of exercise		Benefits of exercise
		Stimulation

I can do it	Perception of Ability	
Ability to sustain intensity		
Ability to exercise longer than 30 min		
Ability to cope		Ability to cope

From exercising	Achievement	From exercising
Performance accomplishments		Performance accomplishments
Satisfaction		Satisfaction

Thinking about nothing	Focus of Attention	Thinking about nothing
Not aware of time		Not aware of time
Thinking of things outside exercise		Exercise happening without thought or attention
Switching off from body		Association with interoceptive cues
Focused on exercising		

	Anticipation of the End	

Cardiorespiratory	Awareness of Interoceptive Cues	Cardiorespiratory
Temperature		Temperature
Perspiration		Perspiration
Muscular		Muscular
Integration of cues		Integration of cues
Level of fatigue		

Warm up	Perception of Physiological State	Warm up
Steady state/rhythm		Steady state/rhythm
		Getting into rhythm

Appropriateness	Self-monitoring of the Exercise Intensity	Appropriateness
Comfort		Comfort
Preference for intensity		

	Perception of Control	

### Pre-Exercise Affective State

This category described the influence that the women's general affective state before exercise had on their affective response during exercise. The low-active women described themes related to state of mind, positive pre-exercise affective state and negative pre-exercise affective state. Only the negative pre-exercise affective state theme emerged from the high-active women. State of mind characterised comments such as, "I must be in a good place in my head" (10 min, LA, SS) and, "I'm a fairly positive person I think most of the time" (5 min, LA, P) which had a positive influence on affective responses. Feeling positive pre-exercise reflected comments like, "I think overall I feel good because I've had a good day" (5 min, LA, P). For the low-active women, feeling positive pre-exercise was, in part, due to their motivation for exercise, "I'm really feeling that I want to get back into my exercise, I looked forward to coming here today" (10 min, LA, P). Alternatively, negative pre-exercise affective state seemed to lower affective responses during exercise in both the high-active and low-active women. This comprised feeling tired, *"*I'm feeling very tired just generally before I got on the treadmill so that would take me lower [on Feeling Scale]" (5 min, LA, P) and having low levels of motivation, "I probably wasn't actually looking forward to doing this tonight necessarily, so I kind of was coming with a little bit of, not dread but kind of, do I really want to do this?" (5 min, HA, SS). The influence of pre-exercise affective state was summed up, "I think it all depends on how you start, if you start feeling good then I keep that, but if I start feeling unmotivated then it's very difficult to improve" (10 min, LA, P).

Quantitative research has shown that affective state prior to exercise can influence the extent to which exercise will improve affective state [e.g., [45,46]]. Our results showed that the influence of pre-exercise affective state (whether positive or negative) seemed to have its main influence in the first 10-15 min of the exercise session. Potentially, once the women started exercising their negative pre-exercise affective state improved. It was interesting to see the influence of exercise motivation impacting on pre-exercise affective state. This may suggest that if individuals arrive to an exercise venue motivated and enthusiastic about exercise then this is one factor that can contribute to the affective response to that exercise session being positive.

### Outcomes from Doing Exercise

This category described the immediate outcomes and anticipated outcomes that the women felt as a consequence of the process of exercising. For both the high-active and low-active groups, this category comprised themes relating to doing exercise, immediate consequences of exercise, enjoyment of exercise and longer term benefits of exercise. Doing exercise reflected that the women felt good because, "I'm actually doing something physical, rather than putting something off" (10 min, LA, SS) and because of the feelings associated with just moving, "It's just moving my body you know, I sit around all day at a desk and it's just nice to move" (5 min, HA, SS).

The women described the consequences of exercise as feeling more awake, more relaxed, more invigorated, happier, energized, generally feeling better and providing a time out from life's worries. For example, "just the feeling that you're waking up more, you've got air in your lungs or your body's moving, everything's sort of functioning" (5 min, HA, SS). The high-active women also described a theme relating to stimulation, "at the beginning...there was a lot of emphasis on feeling stimulated and kind of that rush I get from beginning exercise" (30 min, HA, SS).

Both the high-active and low-active women stated that their affective response was "because I'm enjoying what I'm doing, I'm quite liking the walk" (20 min, HA, P); "cause this feels ok, it feels actually quite enjoyable" (5 min, LA, SS). The high-active women also explained that they enjoyed the exercise because "you know it's doing you good" (25 min, HA, P); for "the challenge of making myself do it" (5 min, A, SS) and because it was an enjoyable intensity. Motivation research suggests that exercising because of the inherent enjoyment gained is important for long term exercise behaviour [47] and is linked with more positive psychological outcomes, including positive affective responses [48]. Thus, the fact that the low-active women in particular were experiencing enjoyment is a positive outcome.

As well as the immediate consequences of exercise, the women were thinking about the longer term outcomes of continued exercise participation and this contributed to affective responses. For example, "I always like to think that I'm feeling healthy on the inside as well as the out and they always say that, you know, that if you're exercising that's good for you" (10 min, LA, P). This supports previous research [12] in showing that individuals evaluate the longer term health and fitness benefits that exercise can provide to generate an affective response. This result is not surprising given that exercise, particularly in the adoption stage, is a goal directed activity and the sense of whether exercise is worthwhile comes from the achievement of such goals [49,50]. Fredrickson [51] has also proposed that the affect that accompanies the outcome of an experience is an indicator of whether the goal directed activity has been perceived as worthwhile. This study also adds that the immediate consequences of exercise are also important to the value of the activity and hence the generation of the affective response.

### Perception of Ability

This category described how the women's thoughts relating to their exercise ability influenced their affective responses. This category was particularly prevalent for the low-active women with perceptions of ability being determined from thoughts of, 'I can do it', ability to sustain intensity, ability to exercise longer than 30 min, and having an ability to cope. For the high-active women only the ability to cope theme emerged. 'I can do it' related to the perception of being able to complete the exercise, "I know I'm a good walker so I know that I can do this" (5 min, LA, SS), even when it was harder than normal, "I was thinking this is a wee bit harder than what I would be if I was just going for a normal walk...but now I can do this" (20 min, LA, P). This helped the women to know that it wasn't going to have a negative impact on their self-esteem, "I feel like this is manageable so you know I'm not going to kill myself, I'm not going to go away from here thinking bad thoughts about myself" (10 min, LA, SS). The women specifically commented that being able to sustain the intensity influenced affective responses, "I still feel fine I know that I can continue...like my body can keep up with the pace, I don't feel like I'm struggling" (20 min, LA, P). While, at the end of the exercise session, feeling able to continue exercising after the 30 minutes was finished was a positive influence, "Yeah I'm feeling good, felt like I could have continued if I needed to without a problem" (30 min, LA, SS). Both the high-active and low-active women described having an ability to cope, "I'm not down in the [feeling] bad area because I feel like I'm coping with this exercise and its not too difficult" (30 min, LA, SS).

Particularly for the low-active women, feeling confident in being able to exercise was important to feeling that exercise was pleasant. Similar to Rose and Parfitt [12], participants became more confident as the exercise continued and they knew they could successfully accomplish the task. Encouragingly, after the initial five minutes, there were very few comments which suggested individuals doubted their ability to continue. For exercise professionals this is heartening because if an exercise intensity around VT is prescribed or individuals are allowed to self-select their intensity these intensities will be perceived as achievable and should support the development of self-efficacy.

The influence of self-efficacy on acute affective responses and on exercise motivation in general is well recognised [52], consequently, we expected that a theme related to perceptions of ability would emerge. Fredrickson [51] proposed that affect experienced during an experience (particularly the peak affect) carries meaning relative to the individual's personal capacity to achieve and cope with the exercise. This theme demonstrates the influence of this cognitive appraisal of ability and coping on the affective response experienced during exercise. The expected differences between the high-active and low-active women emerged with the low-active being more preoccupied with thoughts about their exercise abilities. The high-active women seemed to hold strong efficacy beliefs and did not question their ability to exercise and consequently, this theme played a less prominent role in determining their affective response. These results reiterate the need for exercise prescriptions which support the new exercisers perceptions of confidence, both to increase motivation for exercise and to ensure pleasant affective responses occur.

### Achievement

This category characterized the achievement that both high-active and low-active women felt from doing exercise, from what they achieved during the exercise and of the satisfaction this achievement provided. A sense of achievement was gained from a number of sources. At the end of the 30 min it was gained from having done some exercise, "if I achieved my exercise in the day I'll feel good for the rest of the day so its kind of like, 'ah tick I've done that, I don't have to fit that one in now"' (30 min, HA, P) and from "giving yourself a good blow out, you feel better" (30 min, HA, SS). During exercise, achievement was gained from having successfully exercised at a higher intensity or for longer than normal, "I'm feeling a sense of accomplishment that I've kept it up at probably a faster pace" (15 min, HA, P), "a feeling of achieving 'cause I've gone longer" (20 min, LA, P) or because, "I achieved my goal which I didn't think I could achieve so that made me feel good" (30 min, HA, SS). For low-active women there was a sense of achieving something they had not done in a while or they hadn't realized they could achieve, "I think it's the just the fact that I'm doing something that I haven't done before and I'm succeeding at it" (15 min, LA, SS); "it's almost like an accomplishment, 'yeah I can do it, good' and that makes me feel better" (15 min, LA, SS) and also from the fact that they got around to exercising, "I would like to do things that are good for my body but..um..there's lots of reasons why I'd put it on the back burner, so if I get around to it I feel good about that" (10 min, LA, SS).

Rose and Parfitt [12] discussed the influence of sense of achievement on affective responses through their outcomes of exercise theme because participants only described the sense of achievement felt at the end of the exercise. However, participants in this study described a sense of achievement not simply as an outcome of exercise but more related to their performance accomplishments during the exercise session and even the achievement of having successfully negotiated the barriers and participated in some exercise. Self-Efficacy Theory [53] would propose that this sense of achievement and mastery will reinforce the individual's confidence in their abilities to exercise.

### Focus of Attention

This category described, on what and where, the women were focusing their attention during the exercise. Stevinson and Biddle [54] proposed that attention during running could be characterised according to two dimensions: associative/dissociative processes and external/internal focus relative to the body. Emergent themes reflected these different attentional strategies. The themes differed between the high-active and low-active women. The high-active women explained their affective responses were positively influenced in the first 15 min because the exercise happened without thought or attention, "it just reaches that state where I don't have to think about it, it's just like I'm on automatic pilot now so I can kind of keep going forever" (15 min, HA, P). However, as the exercise went on, the women associated more with interoceptive cues, "as exercise kind of goes on...you know it takes it's toll physically in terms of sweating you know and greater exertion, then yeah I guess I focus on those things" (20 min, HA, P). This was particularly the case when the participant was not experienced with running, "I think it's just because I'm not used to it, [with] running I'm kind of constantly analyzing how every part of my body's feeling at any one moment (25 min, HA, P).

According to Stevinson and Biddle's [54] terminology these high-active women show elements of the associative/internal strategy and, in line with previous research, show that as the intensity becomes more effortful (e.g., as intensity increases or exercise duration continues) attention becomes more associative [55]. In the initial stages, the women reported exercise happening automatically which could reflect an external dissociative strategy or it maybe a completely separate form of attentional coping that has yet to be formalised.

The low-active participants described a different focus of attention with themes describing deliberately thinking about things outside of exercise, " it helps me if I'm not thinking about the exercise, if I'm thinking of other things" (20 min, LA, SS); switching off from body, "I switch off from it [body] and I think that means that I can push myself further because I do switch off from it" (15 min, LA, P) and being focused on exercise, "I feel I have to concentrate on just walking" (10 min, LA, P). The first two themes demonstrate deliberate dissociative strategies being employed by members of the low-active group. Attention is being focused away from the exercise and the body (external) or towards more problem solving activities (internal). Stanley, Pargman and Tenenbaum [56] have shown that the use of dissociative strategies led to a cycling task being perceived as 'easy'. They suggested that adopting these strategies may make the task seem less daunting. Consequently, our less experienced low-active group may naturally adopt these strategies as a coping mechanism.

Both groups reported thinking about nothing, "I'm just walking along not really thinking about anything much, just nothing really. It's quite good to be thinking about nothing" (10 min, HA, SS), and not being aware of time, "I'm thinking... that 15 minutes has gone amazingly quickly" (15 min, HA, SS). Both of these themes were influenced by the exercise intensity, "I try to think of nothing, until it [exercise] becomes extremely hard and then your brain is driven by what the body can do and how long it can hold up" (10 min, LA, SS) and, "it wasn't as hard like I didn't feel it in my chest you know, pounding and pounding yeah, so it's...you know 'oh gosh is it the end already?" (30 min, LA, P). Again, these themes demonstrate the use of dissociative strategies. Since individuals were able to use dissociative strategies this suggests that they did not interpret the exercise as being too intense (and that the interoceptive cues were not dominating) such that dissociation was not possible.

Previous research has shown that the individual's focus of attention during exercise influences affective responses [12,14,22,23,29]. Specifically, having a dissociative focus improves affective responses, while having an associative focus is associated with declining affective responses. Our results showed that in the first 15 min the focus of attention was largely dissociative, but in the latter stages of the exercise focus of attention became more associative as individuals described becoming more preoccupied with the physiological symptoms associated with the exercise. Therefore, in the latter stages of exercise, it may prove beneficial to implement strategies which help individuals to continue to dissociate their attention away from the exercise so that they continue to view the exercise as pleasant. Since our study was conducted in the lab environment individuals may be more likely to associate with their physiological symptoms than if they were in a more stimulating environment where there are more external distractions on which to focus attention. Conducting this research in a more externally valid exercise environment should be the subject of future research and will likely result in additional cognitive factors emerging.

### Anticipation of the End

Towards the end of the exercise, both the low-active and high-active women stated that knowing that they were close to the end had a positive influence on their affective responses, "I think it was probably also knowing that it was..ahh..that I would probably be ending soon yeah that was kind of in my mind so that was kind of a you know boosting up" (30 min, HA, P). Baden et al. [57] investigated the influence of anticipation on Feeling Scale responses in well trained participants during exercise by manipulating whether exercise duration was known (20 min), unknown, or unexpected (told they would exercise for 10 min but at 10 min were asked to exercise for a further 10 min). Their results showed that affect fell more steeply from 10 to 11 mins in the unexpected condition and remained lower at 14 min compared to the other two conditions. Although this study does not demonstrate the increased positivity that was experienced knowing that the end was near, it demonstrates that when individuals think they are at the end of the exercise and they are asked to continue, their affective response is negatively affected. Therefore, encouraging exercisers to set a duration goal for their exercise session may lead to more positive affective responses towards the end of exercise.

### Awareness of Interoceptive Cues

To decide how they were feeling, both high-active and low-active women made reference to the physiological symptoms they were experiencing as they exercised. These interoceptive cues were grouped into themes comprising cardiorespiratory, temperature, perspiration and muscular cues and a theme representing an integration of cues. The low-active women also described fatigue as a separate theme. Affective responses were more pleasant when participants had a positive interpretation of the increase in heart rate and breathing, "can feel the old heart pumping it feels good" (10 min, HA, SS); when the cardiorespiratory cues were not too intense, "feeling good because I'm not puffing too much and my heart rate's not too high as far as I'm concerned" (5 min, HA, SS); and when the interoceptive cues provided information about the benefits of exercise, "I do feel a little bit breathless at time, so it feels as though it's doing me some good" (20 min, LA, SS).

Particularly in the low-active women there seemed to be a temperature at which they felt good but as they got hotter then this had a negative impact on affective responses, "I think the drop to [feeling] 'fairly good' was because I'm starting to feel very hot" (30 min, LA, P). The women also differentiated between feeling warmer and perspiring. Most women viewed their perspiration positively and again as an indicator that exercise was of benefit, "when you actually perspire somehow that makes me think that I have done some you know done some good for my body" (15 min, LA, P). However, one low-active participant viewed their perspiration as uncomfortable, for another it caused her to feel a little self-conscious and for another it caused her to feel flustered, "I suppose I feel a wee bit flustered...I'm not one that has really pushed myself physically over the years so I suppose I don't like to be somebody that's sweating" (20 min, LA, P).

Muscular cues were more prevalent in the low-active women, "just probably the reaction from my legs you know that you can feel it...not such a pleasant experience" (15 min, LA, SS). The high-active women seemed to comment on muscular cues only if they had a niggling complaint. The low-active women also commented on their level of fatigue as an interoceptive cue, "emotionally I feel really good but physically I was getting a bit tired" (30 min, LA, P).

Although the women commented separately on each of the different interoceptive cues, there were also times when they would consider an integration of these cues to inform their affective response,

I am feeling quite hot..ah..which is quite early in the run for me to be feeling so hot so I'm not finding that that comfortable. But I've got my breath back and my muscles aren't hurting anymore in my legs so hence why everything's improved (10 min, HA, P).

To decide on the cause of their affective response two women explained,

"it's almost like a systems approach like how's my breathing, how's my heart feeling, am I sweating too much, do I feel uncomfortable, do my legs hurt...yeah. There's no pain, no breathlessness, heart feels like it's pumping ok, don't feel too sweaty ok good" (20 min, LA, SS).

These results illustrate the importance of the physiological symptoms that exercise produces in the generation of affective responses. Interestingly, there was no specific mention of the role of physiological symptoms in the results of Rose and Parfitt [12]. This is likely due to the different methodologies employed. Rose and Parfitt [12] used a recall protocol after exercise, at which time the physiological symptoms would be dissipating and individuals may not remember or associate the influence they had on their during exercise affective response. However in this study, the protocol analysis captures what influenced the women's affective response while these physiological symptoms were being experienced and as a result they were recognised as salient to their affective response.

Our results show that the individual's interpretation of their physiological symptoms was important for their effect on affective responses. For example, the women felt more pleasant when they had a positive interpretation of their increased heart rate and breathing which also provided an indictor that the intensity was beneficial. Alternatively, the women felt less pleasant when the cues were interpreted more negatively. For example, when increased temperature and perspiration led to feelings of self-consciousness and a loss of comfort. The DMM predicts that at intensities around VT the interplay of both psychological appraisal and interoceptive cues will determine affective response [15,16]. Our results suggest that around VT, the influence of interoceptive cues on affective responses depend on how they are interpreted. If they are interpreted positively and as not being overwhelming then individuals will feel pleasant. However, when they are interpreted negatively then affective responses suffer. Importantly, our results show the individual nature of this interpretation because some individuals could interpret the cue positively while others could not. However if the intensity was to continue to increase above VT, then interoceptive cues will have a more direct influence on affect as the DMM predicts. From a practical perspective, changing the individual's interpretation of their physiological symptoms to become more facilitative may be one avenue to increase the pleasure experienced from exercise at this intensity.

### Perception of Physiological State

This category characterized the women's perception of the physiological state of their body. It is distinct from the previous theme, Awareness of Interoceptive Cues because it was not simply the symptoms they were experiencing which impacted on affective responses, but rather how those cues influenced their perception of their body's physiological state. In the first half of the exercise session, the women felt that they were warming into the exercise and that had a positive effect on their affective responses, "I was just getting warmed up yeah, body's a bit looser and I'm starting to get into it" (5 min, HA, SS). In the latter stages, comments were more in line with being in steady state, "It's more kind of into that steady state of exercising, that's really what's making me feel good" (15 min, HA, SS), or feeling in a rhythm, "I feel a bit more sort of into the rhythm of it and a bit more sort of comfortable" (15 min, LA, P). The high-active women also talked about trying to get into a rhythm which lay between having warmed up and reaching steady state, "well it doesn't feel quite right until you get into the rhythm of it, so it's just finding the rhythm that works on a certain day" (20 min, HA, SS). These results suggest that affective responses were positively influenced in the initial stages of the exercise because of the sense of warming into the exercise, while in the later stages of the exercise, affective responses were positively influenced because of the feeling of being in a steady state or rhythm.

### Self-monitoring of the Exercise Intensity

This category described how the women self-monitored the appropriateness and comfort experienced from exercise intensity to inform their affective response. Affective responses were positively influenced when the intensity was perceived as being at an appropriate level, "this is a great pace and I don't know what the level of exertion is, it just seems to be right" (5 min, HA, P). Alternatively, "when it started to get a bit harder I didn't feel as good about it" (25 min, HA, SS). The intensity was considered appropriate because, "I can feel I'm sort of working a wee bit but you know I don't feel overwhelmed and I feel like it's quite pleasant just doing this" (5 min, LA, SS). It was perceived as inappropriate when it was set too low (in the self-selected condition), "I should have taken it to the max... yeah I think I should have really tried to go a lot harder than I did" (30 min, HA, SS), and when it was set too high (in the prescribed condition), "if I were choosing my pace I wouldn't be running quite this fast today, I might be another day, but not today" (5 min, HA, P). When the intensity was appropriate it was described as comfortable, "feel quite comfortable, not working overly hard...not out of breath sort of gasping for air um feel my hearts working at a reasonable pace but not too much, muscles feel relaxed, fairly comfortable" (5 min, HA, P).

This category highlighted that the majority of the women felt that the intensity that was prescribed and chosen was appropriate for them. They described the intensity as comfortable and beneficial, but not overwhelming and these factors contributed to a positive affective response. It is likely that this positive interpretation of the intensity will have contributed to the strong perceptions of ability (described previously) that they felt towards the exercise. Further, it is likely that the women's interpretation of the interoceptive cues will also have influenced their perceptions of how comfortable and appropriate the intensity was. Ekkekakis and colleagues [5] have discussed that individuals who are more predisposed to tolerate discomfort are less likely to experience a decline in affective valence. They have also shown that individuals have a predisposition to prefer certain exercise intensities and that this predisposition significantly predicts self-selected exercise intensities [19]. These concepts of preference and tolerance have been shown to correlate with affective responses during exercise at VT (r = .58 & .47 respectively). As a consequence, it would be expected that when an imposed intensity matches that preferred level or in the context of this study, is perceived as 'appropriate' then affective responses will be positive.

We showed that the prescribed intensity was perceived as inappropriate when it was felt to be too hard. Recently, Lind et al. [18] have shown less positive affective responses result when exercise is prescribed just 10% above that which individuals self-select. Therefore, compromised affective responses result when a prescribed intensity is not in line with personal preference. During our self-selected condition, affective responses were less positive when the women struggled to find an appropriate intensity, e.g. when the intensity was set too low or because they were unfamiliar with the mode of exercise. The need for practice in being able to self-regulate intensity has been discussed previously for low-active individuals [58].

### Perception of Control

Unique to the low-active women was a theme describing the feeling of being in control of the exercise. The influence that being in control had on affective responses differed depending on the exercise condition. During the self-selected intensity the women stated that being in control positively influenced affective response because it was empowering and because, "in my mind I know that I can definitely achieve the 30 minutes because you've given me control over making it slower if I felt that I couldn't manage" (15 min, LA, SS). Alternatively, during the prescribed intensity condition, positive affective responses resulted because individuals had given up control of the exercise intensity, "it's a pleasurable state because I'm not, I don't have to decide when to stop, that's already been decided" (10 min, LA, P);

"I think it's got to do with you setting the scales for me, I'm too timid to see whether I can do that because I don't want to say 'ah no I can't' and then fail. Whereas if you did it...it's also about showing me that I can do it" (20 min, LA, P).

Perceptions of control have been suggested as an important factor in determining affective responses particularly when intensity is self-selected [12,18,59] and as shown in our results, can result in feelings of empowerment and confidence. Recently, Vazou-Ekkekakis and Ekkekakis [59] found that energetic arousal and interest/enjoyment was greater following an exercise session with higher autonomy compared with a more controlling condition, however, there were no differences in the pleasure-displeasure dimension of affect during exercise. Our results showed that in the prescribed condition having someone else control the intensity also resulted in positive affective responses with the individuals feeling motivated, challenged and having greater awareness of their exercise capabilities. It could be argued that in this prescribed condition, the women were still exerting their autonomy because they were happy to give up control of the intensity and for others to regulate their exercise. Therefore, the influence of control on affective responses may not be due to the objective sense of "being controlled" that is intended during a prescribed intensity condition, but whether or not the individual is willing to give up control of their behaviour to someone else. The extent to which perceptions of autonomy and control influence affective responses to exercise should continue to be the subject of future research.

## Conclusion

The purpose of this study was to explore the cognitive factors that influence affective responses to exercise to begin to understand why affective responses to exercise of the same intensity differ between individuals. Support for the hypothesis that affective responses to exercise influence exercise adherence is emerging [2,60]. Consequently, if we understand how positive affective responses are generated then the exercise experience can be constructed in such a way as to ensure a pleasure response and therefore, have a positive effect on continued participation [3,12]. Backhouse et al. [3] believe, "affective responses to exercise are subject to multiple influences, including the physiological and psychological characteristics of the participants, the physiological demands of the exercise stimulus, the physical and social environment, and a multitude of situational appraisals, all of which form a complex web of interactions" (p. 511). Our results illustrate this complex interaction. The core categories that emerged provide an insight into the cognitive appraisal process that is necessary to make it more likely that a positive affective response will be experienced during exercise. Women need to perceive they are capable of completing the exercise asked of them (perceptions of ability category), be somewhat motivated to participate in exercise (pre-exercise affective state category), consider that they are experiencing, and will obtain, important benefits from the exercise (outcomes from doing exercise category), which include a sense of goal achievement (achievement category). It is important that their attention is focused away from the symptoms of the exercise itself (focus of attention category) and that support is provided for the individual's autonomy (perception of control category). Furthermore, the physiological symptoms of the exercise stimulus will have a positive influence on affective responses when women can perceive them as facilitative rather than debilitative (awareness of interoceptive cues category), when they feel comfortable and not overwhelmed (self-monitoring of the exercise intensity).

Our findings both confirm and extend the findings of Rose and Parfitt's [12] initial research investigating the cognitive appraisal process and are consistent with expectations derived from previous quantitative research, e.g., role of attentional focus [e.g., [14,54]] and perceptions of ability [e.g., [20]]. The influence of how individuals felt prior to exercise on affective responses during exercise was not reported by Rose and Parfitt, nor was the influence of the immediate benefits experienced during exercise. As previously discussed, this may reflect the different methodologies employed between the two studies; post-exercise recall versus verbal reports during exercise. With retrospective recall, it is likely that individuals do not verbalise everything that they were thinking during that time. Some information may have a stronger representation in memory and therefore be easily recalled, while other information which was only attended to briefly during the exercise maybe forgotten. The use of protocol analysis as a method for data collection was a strength of this study because the information was obtained as it was being processed and may explain why new themes emerged from this study that were not verbalised by the participants in Rose and Parfitt's research. However, protocol analysis is not without its own potential problems. Difficulties can occur with participants being unable to verbalise every thought that comes into their mind within a particular time frame [25]. Individuals can find putting words to their thoughts problematic, indeed, the women in this study differed in the depth of information that they could provide in relation to their thoughts. Furthermore, some women were exercising at an intensity where it may have been difficult to talk at length because of the increased breathing rate [29]. A further methodological consideration for the use of verbal reports is that since we asked the women to tell us how they were feeling and the factors that influenced their affective responses during the exercise, this may have interfered with the natural generation of an affective response. As a result, this may have changed the explanations for the affective response provided by the women that would have been elicited in a more natural environment. Therefore, although our results are comprehensive they cannot be deemed a full account of the cognitive appraisal without further confirmation.

It was interesting to find that the core reasons that contributed to the affective response in high-active women were similar to those of low-active women. However, there were some subtle differences in the themes that comprised those core categories. For example, the high-active women did not question their ability to exercise at the required intensity, presumably their increased exercise experience meant that they had greater confidence. The category related to pre-exercise affective state highlighted that, although our low-active group were not currently exercising, they were clearly motivated to try and increase their physical activity behaviour and this was reflected in having a positive frame of mind prior to participating in exercise. Exercise studies such as this one are limited in that individuals with little interest in exercise are unlikely to volunteer. Therefore, the thought processes that the women had that impacted on their affective responses during the exercise should be interpreted in light of these characteristics. If a sample of resistant exercisers had been recruited then the results may have been different. Further considerations should be taken into account when interpreting these results. Since this research was focused on women, these results are only relevant for women. The exercise was performed in a laboratory setting, if the exercise had been conducted in a different social environment, e.g., outdoors, then other cognitive factors may have influence on affective responses. Future research should endeavour to identify the cognitive appraisal process in men and the additional cognitive factors that influence affect in more externally valid exercise settings.

From an exercise prescription and motivational perspective our results provide some tentative guidelines on how exercise professionals could help individuals experience positive affective responses from exercise. This could occur through restructuring exercise sessions to allow self-regulation of exercise intensity or ensuring the intensity prescribed is within individual's capabilities and perceived as comfortable. This may seem obvious, but as Lind et al. [18] have shown, setting an intensity just 10% above that which is preferred can have a negative influence on affective responses. Recent research has begun to explore the use of psychological strategies commonly applied in sport in the exercise context. For example, Stanley and Cumming [61] have shown that post-exercise affect is significantly more positive when individuals use 'enjoyment' imagery before exercise, compared to individuals who use 'technique' or 'energy' imagery. While Gammage et al. [62] have shown that self-talk is used frequently during exercise with its main functions being motivational (for mastery of skills, to control arousal and maintain drive to exercise) and cognitive (to focus on technique and to encourage more effective workouts). Therefore, imagery and/or self-talk strategies could be used to help build motivation for the exercise itself and to support positive perceptions of ability. Additionally, individuals could be helped to cognitively restructure their thought processes throughout the exercise. For example, by ensuring that individuals adopted a dissociative attentional focus, that they were encouraged to view their increased heart rate, breathing etc in a facilitative manner, to focus on their personal achievements in relation to exercise and lastly, to focus on the positive outcomes that were being achieved through exercise. Exercisers may also benefit from knowing how long the exercise session will last as this will provide a sense of achievement from reaching it, and will also help the individual to be psychologically prepared for the specific duration. The next step for research may be to focus on the manipulation of these variables and implementation of certain psychological strategies to see if a positive affective response to exercise can be facilitated. Based upon previous research, we may anticipate that positive effects will be more apparent at intensities around and below an individual's ventilatory threshold where affective responses have been shown to be influenced by cognitive processes.

## Competing interests

The authors declare that they have no competing interests.

## Authors' contributions

ER & GP conceived the initial idea for the study, developed its methodology and conducted the data analysis. ER carried out the data collection and wrote the first draft of the manuscript. GP provided comments to redraft the manuscript. Both authors read and approved the final manuscript.
